# Multimodal MRI links gray matter volume loss and temporal lobe susceptibility to CSF immunological profiles in Guillain–Barré syndrome

**DOI:** 10.3389/fimmu.2026.1942893

**Published:** 2026-09-03

**Authors:** Wei Wang, Xin Wang, Siyu Fan, Jianru Sun, Jie Hu, Shugang Cao, Jing Du, Lili Tang, Ping Qu, Hong Chen, Guankai Zhao, Zhongyuan Hu, Xiaokun Qi, Dai Zhang, Jun Zhang

**Affiliations:** 1 Department of Neurology, The Second Affiliated Hospital of Anhui Medical University, Hefei, China; 2 Anhui Medical University, Hefei, China; 3 Department of Neurology, The First Affiliated Hospital of Anhui Medical University, Hefei, China; 4 The First Affiliated Hospital of USTC (Anhui Provincial Hospital), Hefei, China; 5 Collaborative Innovation Center of Neuropsychiatric Disorders and Mental Health, Hefei, China; 6 Anhui Province Key Laboratory of Cognition and Neuropsychiatric Disorders, Hefei, China

**Keywords:** magnetic susceptibility, cerebrospinal fluid biomarkers, Guillain-Barré syndrome, hippocampus, neuroinflammation, QSM, VBM

## Abstract

**Background:**

Guillain–Barré syndrome (GBS) is an acute immune-mediated disease of the peripheral nervous system, with accumulating evidence of increased levels of multiple inflammatory factors in the central nervous system. Previous studies have suggested that inflammatory factors may mediate changes in brain structure and function by activating glial cells and altering the expression of iron-regulatory proteins. However, direct imaging evidence supporting these alterations in GBS remains limited. Therefore, based on multimodal magnetic resonance imaging, this study preliminarily investigated potential changes in gray matter structure and iron metabolism in GBS, and further explored their associations with fluid biomarkers.

**Methods:**

A total of 25 GBS patients and 39 healthy controls were enrolled and underwent multimodal brain MRI examinations. Voxel-based morphometry (VBM) was used to assess differences in whole-brain gray matter volume (GMV), while quantitative susceptibility mapping (QSM) was used to evaluate tissue magnetic susceptibility. For exploratory analyses, mean GMV and magnetic susceptibility values were extracted from significant whole-brain clusters and correlated with blood and cerebrospinal fluid (CSF) indicators in GBS patients.

**Results:**

GBS patients exhibited reduced GMV in the bilateral hippocampal–parahippocampal–amygdala regions and increased magnetic susceptibility in bilateral temporal regions. Exploratory analyses revealed negative associations between GMV in the left hippocampal cluster and several CSF markers involving IgA, IgG, and the CSF-to-serum total protein ratio.

**Conclusion:**

GBS patients exhibit potential alterations in brain GMV and magnetic susceptibility-related tissue properties. These findings suggest that GBS may be accompanied by changes in central brain tissue status, and gray matter abnormalities in the limbic system may be associated with alterations in the CSF immune-metabolic microenvironment.

## 1 Introduction

Guillain–Barré syndrome (GBS) is an acute immune-mediated polyradiculoneuropathy and one of the leading causes of acute flaccid paralysis worldwide. It primarily affects the peripheral nervous system, including nerve roots and peripheral nerves, and typically presents with rapidly progressive limb weakness, areflexia, sensory symptoms, and autonomic dysfunction (1, 2). GBS was relatively uncommon before 2020; however, its global burden has increased substantially in recent years. A Global Burden of Disease (GBD) 2021-based analysis reported that the estimated number of individuals affected by GBS in 2021 was approximately twice that in 2020. The age-standardized years lived with disability rate increased sharply during 2019–2021, with an estimated annual percentage change of 70.35% (3). Despite immunotherapy and supportive care, approximately 30% of patients may require mechanical ventilation, while 3–10% may die and many survivors experience persistent disability, pain, or fatigue (4, 5). Therefore, for patients with a high risk of poor prognosis, a deeper investigation into the underlying mechanisms is essential.

Although GBS is generally regarded as an acute immune-mediated disorder of the peripheral nervous system, accumulating clinical, pathological, and neuroimaging evidence suggests that its disease process may not be strictly confined to the peripheral nervous system. Patients with GBS may present increased intracranial pressure, posterior reversible encephalopathy syndrome, altered consciousness and other encephalopathic features (6–9), all of which indicate potential central nervous system involvement. Moreover, Maier et al. reported mononuclear inflammatory infiltrates in the spinal cord, medulla oblongata, pons, and midbrain of patients with GBS, together with microglial activation involving the spinal cord, brainstem, ventricular system, and central canal of the spinal cord (10). Therefore, while GBS remains primarily a peripheral nervous system disorder, these findings raise the possibility that central immune activation or secondary central responses may contribute to the complex clinical manifestations observed in some patients. Meanwhile, previous studies of peripheral nerve injury have shown that disruption of afferent and efferent communication between peripheral nerves and the central nervous system may induce cortical plasticity, characterized by neurochemical alterations, adaptive changes in excitatory and inhibitory neuronal systems, and reorganization of somatosensory and motor cortical regions (11–15). These observations provide a mechanistic link between peripheral nerve injury or inflammation and broader central nervous system responses. Accordingly, it is biologically plausible that GBS patients may exhibit potential brain structural or microstructural alterations. However, neuroimaging in GBS has traditionally been used mainly to exclude central nervous system mimics or to identify nerve-root abnormalities, whereas systematic quantitative brain MRI assessments remain scarce (5, 16). Furthermore, whether these potential brain alterations are associated with disease-related clinical, inflammatory, or biochemical characteristics remains unclear.

Prior studies have shown that higher levels of peripheral inflammation are associated with lower gray matter volume (17–19), and inflammatory cytokines may further aggravate iron dyshomeostasis by activating microglia and astrocytes, altering the expression of various iron-regulatory proteins, and promoting oxidative stress (20, 21). Although alterations in multiple blood and cerebrospinal fluid (CSF) inflammatory factors have been reported in GBS (22), there is no clear evidence demonstrating brain volume or iron metabolism alterations in GBS. Moreover, whether changes in inflammation-related factors are associated with region-specific brain volume abnormalities or iron metabolism disturbances, and whether these brain alterations may be related to disease-related clinical manifestations, remains unclear. Voxel-based morphometry (VBM) enables whole-brain assessment of regional gray matter volume differences (23), whereas quantitative susceptibility mapping (QSM) quantitatively reflects tissue magnetic susceptibility and is sensitive to changes related to iron deposition, myelin integrity, calcification, and other microstructural tissue components (24). Therefore, the combined application of VBM and QSM may allow the assessment of GBS-related brain alterations at both the macroscopic gray matter structural level and the susceptibility-related microstructural level.

In the present study, we employed a combined VBM and QSM approach to investigate underlying alterations in gray matter volume and magnetic susceptibility in patients with GBS, and to further explore their associations with clinical and body-fluid biomarkers. By integrating macroscopic structural and susceptibility-related microstructural information, this study aimed to provide additional neuroimaging evidence for potential central nervous system involvement in GBS and to offer new insights into the interaction between peripheral immune-inflammatory processes and brain alterations.

## 2 Methods

### 2.1 Participants

Patients with GBS and healthy controls (HCs) were recruited from the Department of Neurology of the Second Affiliated Hospital of Anhui Medical University. GBS patients were diagnosed by experienced neurologists according to the Chinese guidelines for diagnosis and treatment of Guillain–Barré syndrome 2024 (25). Patients with GBS included those with the classic acute inflammatory demyelinating polyneuropathy (AIDP) as well as other specific subtypes, as detailed in the diagnostic criteria. Additional inclusion criteria were: (1) age between 18 and 75 years; (2) time from disease onset to hospitalization ≤ 6 weeks. The diagnosis and subtype classification were jointly determined by two neurologists with more than 10 years of clinical experience in neurology. Any disagreement was resolved by consultation with a senior neuroimmunology specialist. All MRI scans were reviewed by experienced neuroradiologists to exclude specific structural lesions and ensure adequate image quality. All researchers involved in clinical assessments received standardized training before data collection. The exclusion criteria for GBS were as follows: (1) a history or current diagnosis of diseases that may affect the structure or function of the central nervous system, including acute hemorrhagic or ischemic stroke, intracranial tumors, encephalitis, severe traumatic brain injury, multiple sclerosis, or other inflammatory or demyelinating diseases of the central nervous system; (2) clinically suspected or confirmed disorders that could cause acute flaccid paralysis or acute limb weakness other than GBS, such as myasthenic crisis, myelitis, acute spinal cord diseases, or metabolic neuropathy; (3) severe hepatic or renal dysfunction, severe cardiopulmonary insufficiency, active infection, or other severe systemic diseases that could substantially affect systemic inflammatory status, metabolic status, or study assessments; (4) extremely unstable clinical condition, severe respiratory muscle involvement, or impaired consciousness that precluded safe completion of MRI examination; (5) poor MRI image quality or obvious motion artifacts. HCs were recruited during the same period and were overall balanced with the GBS group in terms of age, sex, and education level. HCs were recruited concurrently from individuals undergoing routine health examinations. All HCs had no apparent structural abnormalities on conventional brain MRI and reported no neurological symptoms, including limb numbness, pain, weakness, or other related complaints. Individuals with a history of neurological disorders, such as stroke, Parkinson’s disease, Alzheimer’s disease, or multiple sclerosis, or with a history of psychiatric disorders were excluded. Individuals receiving immunosuppressive agents, anti-inflammatory medications, or corticosteroids were also excluded. Information on vascular risk factors and lifestyle-related factors was collected through clinical interviews and medical records. Hypertension, diabetes mellitus, history of dyslipidemia, smoking status, and alcohol consumption status are summarized in **Table 1**. This study was approved by the Ethics Committee of the Second Affiliated Hospital of Anhui Medical University (approval number: YX2026-102). Written informed consent was obtained from all participants before enrollment. All procedures were conducted in accordance with the Declaration of Helsinki.

**Table 1:** Demographic and clinical characteristics of participants.

Variable	GBS group (n = 25)	HC group (n = 39)	Test statistic	P value
Age, years	60.00 [50.00–68.00]	61.00 [57.00–62.00]	W = 486.50	0.994
Sex, male/female	18/7	27/12	χ^2^ = 0.06	0.813
Education, years	6.00 [6.00–9.00]	9.00 [5.50–12.00]	W = 392.50	0.183
TIV	1443.54 ± 160.37	1465.13 ± 113.56	t = -0.63	0.531
Diabetes, n (%)	6 (24.0%)	13 (33.3%)	χ^2^ = 0.64	0.425
Hypertension, n (%)	11 (44.0%)	15 (38.5%)	χ^2^ = 0.19	0.660
Dyslipidemia history, n (%)	12 (48.0%)	14 (35.9%)	χ^2^ = 0.93	0.336
Smoking status, n (%)				0.709
Never smoker	13 (52.0)	16 (41.0)		
Former smoker	2 (8.0)	5 (12.8)		
Current smoker	10 (40.0)	18 (46.2)		
Alcohol consumption, n (%)				0.719
Never drinker	16 (64.0)	25 (64.1)		
Former drinker	8 (32.0)	10 (25.6)		
Current drinker	1 (4.0)	4 (10.3)		
Disease duration, days	13.0 [7.0–20.0]	—	—	—
Hughes score	3.0 [2.0–3.0]	—	—	—
CSF protein, mg/L	914.0 [618.0–1082.0]	—	—	—
CSF glucose, mmol/L	3.97 [3.59–4.49]	—	—	—
CSF WBC, ×10^6^/L	4.0 [2.0–39.0]	—	—	—
CSF IgG, mg/L	107.0 [66.2–169.0]	—	—	—
CSF IgA, mg/L	8.3 [4.0–16.2]	—	—	—
CSF IgM, mg/L	2.2 [1.1–3.7]	—	—	—
Blood white cell count, ×10^9^/L	7.46 [5.11–8.87]	—	—	—
Blood neutrophil count, ×10^9^/L	4.81 [3.36–6.24]	—	—	—
Blood lymphocyte count, ×10^9^/L	1.42 [1.10–2.11]	—	—	—
Blood glucose, mmol/L	5.10 [4.67–6.05]	—	—	—
Blood total protein, g/L	65.4 [61.6–69.9]	—	—	—
CSF-to-blood glucose ratio	0.73 [0.67–0.83]	—	—	—
CSF-to-serum total protein ratio, mg/g	15.20 [9.03–18.83]	—	—	—

Continuous variables are presented as mean ± standard deviation or median [interquartile range], as appropriate, and categorical variables are presented as n (%). For CSF WBC count, the full observed range was 0–125 × 10^6^/L. Between-group comparisons were performed using the Mann–Whitney U test for age and education years, the independent-samples t-test for TIV, and χ^2^ tests for categorical variables. Fisher’s exact test was used for smoking and alcohol consumption status. Disease duration, Hughes score, cerebrospinal fluid indicators, and blood laboratory indicators were summarized only for the GBS group; therefore, corresponding HC values and between-group statistics are indicated by “—”. GBS, Guillain–Barré syndrome; HC, healthy controls; TIV, total intracranial volume; CSF, cerebrospinal fluid.

Because prior studies providing exact effect-size estimates for VBM and QSM alterations in patients with GBS are limited, sample size estimation was performed based on previous power-estimation studies using G*Power 3.1.9.7 software (Heinrich Heine University Düsseldorf, Düsseldorf, Germany). The results showed that, with a two-tailed α level of 0.05, a statistical power of 0.80, and a large effect size (Cohen’s d = 0.8), at least 26 participants per group were required (26).

### 2.2 CSF and blood collection and laboratory assessment

CSF samples (5–10 mL) were collected under aseptic conditions by lumbar puncture at the L3–L4 or L4–L5 intervertebral space and immediately transported to the clinical laboratory. Peripheral venous blood was collected concurrently for biochemical testing. CSF aliquots were centrifuged at 3500 rpm for 7 min at 4 °C, and the supernatants were analyzed. CSF glucose was measured using the hexokinase method; CSF total protein using the pyrogallol red–molybdate complex colorimetric (PRM) method; CSF IgG, IgA, and IgM using immunoturbidimetry; and serum total protein using the biuret method. Paired CSF and serum total protein measurements were used to calculate the CSF-to-serum total protein ratio. The ratio was calculated as: CSF-to-serum total protein ratio (mg/g) = CSF total protein (mg/L)/serum total protein (g/L).

### 2.3 MR image acquisition

All MRI examinations were performed at the Department of Radiology, the Second Affiliated Hospital of Anhui Medical University, using a 3.0-T MRI system (MAGNETOM Vida, Siemens Healthineers, Erlangen, Germany). Participants were positioned supine with their heads stabilized using foam padding to reduce head motion and were instructed to remain awake and still during scanning. Earplugs were used to reduce scanner noise. In addition to the research MRI sequences, all participants underwent routine brain MRI scanning, including T1-weighted imaging, T2-weighted imaging, fluid-attenuated inversion recovery imaging (FLAIR), diffusion-weighted imaging (DWI) with corresponding apparent diffusion coefficient (ADC) maps, and susceptibility-weighted imaging (SWI). The above-mentioned MRI sequences were evaluated for intracranial hemorrhage, mass lesions, extensive white matter lesions, or other clinically relevant structural brain abnormalities. Among these sequences, DWI and ADC maps were jointly reviewed to exclude acute cerebral infarction.

High-resolution three-dimensional T1-weighted structural images were acquired for voxel-based morphometry analysis. The imaging parameters were as follows: repetition time = 1900 ms; echo time = 2.98 ms; flip angle = 9°; field of view = 256 × 256 mm^2^; number of slices = 176; slice thickness = 1.0 mm; and voxel size = 1.0 × 1.0 × 1.0 mm^3^.

Quantitative susceptibility mapping data were acquired using a three-dimensional multi-echo gradient-echo sequence. The acquisition parameters were as follows: repetition time = 29 ms; echo times = 5.36, 10.19, 15.02, 19.85, and 24.68 ms; flip angle = 15°; field of view = 220 × 220 mm^2^; acquisition matrix = 320 × 320; number of slices = 96; slice thickness = 1.5 mm; voxel size = 0.6875 × 0.6875 × 1.5 mm^3^; bandwidth = 473 Hz/pixel; and GRAPPA acceleration factor = 2.

### 2.4 Image analysis

#### 2.4.1 Voxel-based morphometry analysis

Voxel-wise gray matter volume (GMV) was measured using Statistical Parametric Mapping version 12 (SPM12, https://www.fil.ion.ucl.ac.uk/spm) implemented in MATLAB (MathWorks, Natick, MA, USA), together with the Computational Anatomy Toolbox 12 (CAT12, http://dbm.neuro.uni-jena.de/cat12/). Structural images were processed following the recommended CAT12 pipeline, including segmentation into gray matter, white matter, and cerebrospinal fluid, spatial normalization to Montreal Neurological Institute (MNI) space, modulation to preserve local gray matter volume, and resampling to a 1.5-mm isotropic voxel size (27). Total intracranial volume (TIV) was obtained for each participant. Image quality was assessed using the Image Quality Rating (IQR) provided by CAT12 (28, 29). The modulated gray matter images were smoothed with an 8-mm full width at half maximum (FWHM) Gaussian kernel and used for subsequent whole-brain voxel-wise statistical analyses (30).

#### 2.4.2 Quantitative susceptibility analysis

Quantitative susceptibility mapping (QSM) processing was performed using STI Suite (https://chunleiliulab.github.io/software/), FMRIB Software Library (FSL, https://fsl.fmrib.ox.ac.uk/fsl/), and Advanced Normalization Tools (ANTs, https://stnava.github.io/ANTs/). First, magnitude and phase images from the five echoes were converted into appropriate format and combined into four-dimensional multi-echo magnitude and phase datasets, respectively. A brain mask was then generated from the first-echo magnitude image using the Brain Extraction Tool (BET) in FSL, and the quality of skull stripping was visually inspected. For Siemens phase images, the raw phase values were rescaled to the range of −π to π before QSM reconstruction. Individual QSM maps were reconstructed using STI Suite, including phase unwrapping, background field removal, and dipole inversion. The individual QSM maps were then normalized to the MNI-152 standard space using ANTs. The normalized QSM maps were spatially smoothed with a 3-mm FWHM Gaussian kernel (31–33). Quality control was performed at each major processing step, including QSM reconstruction, image registration, and spatial normalization.

Following the whole-brain VBM and QSM analyses, brain regions showing significant between-group differences were defined as regions of interest (ROIs). For VBM, mean modulated gray matter volume values were extracted from each significant cluster, whereas for QSM, mean magnetic susceptibility values were extracted from each significant cluster. These ROI-based values were then used for exploratory correlation analyses with clinical characteristics and blood or cerebrospinal fluid laboratory indices in patients with GBS, to further investigate whether brain structural and susceptibility-related alterations were associated with disease-related clinical or biological features.

### 2.5 Statistical analysis

Demographic, clinical, and laboratory characteristics were analyzed using IBM SPSS Statistics for Windows, version 26.0 (IBM Corp., Armonk, NY, USA). The normality of continuous variables was assessed using the Shapiro–Wilk test. Normally distributed variables were summarized as mean ± standard deviation, whereas non-normally distributed variables were summarized as median [interquartile range]. Between-group comparisons were performed using independent-samples t-tests or Mann–Whitney U tests, depending on data distribution. Welch’s t-test was applied when the assumption of equal variances was not met according to Levene’s test. Categorical variables were presented as numbers and percentages and were compared using chi-square tests or Fisher’s exact tests, as appropriate. Clinical and laboratory variables available only for patients with GBS were summarized descriptively. A two-tailed P < 0.05 was considered statistically significant.

VBM and QSM analyses were performed to identify brain regions showing significant differences between GBS patients and HCs. Age, sex, and TIV were included as covariates in the VBM analysis, and age and sex were included as covariates in the QSM analysis. Imaging results were corrected for multiple comparisons using cluster-level family-wise error (FWE) correction at P < 0.05 with an initial voxel-level threshold of P < 0.001. Given the relatively small sample size and the potential non-normal distribution of laboratory variables, associations between imaging measures and clinical characteristics, blood indices, and cerebrospinal fluid indices were assessed using Spearman’s rank correlation analysis. Multiple comparisons were corrected using the false discovery rate (FDR) method.

Sensitivity analyses were further performed to assess the robustness of the main imaging findings and ROI–CSF associations. For the imaging analyses, diabetes mellitus and hypertension were additionally included as covariates in the group comparisons, and the analyses were also repeated after excluding patients with GBS who had diabetes mellitus. These analyses were conducted using the same statistical thresholds as the main VBM and QSM analyses. For the ROI–CSF associations, the correlation analyses were repeated after excluding four patients with CSF WBC counts >50 × 10^6^/L (54, 55, 66, and 125 × 10^6^/L), leaving 16 patients for analysis. The same Spearman’s rank correlation analysis and FDR correction procedure as those used in the primary ROI–CSF analyses were applied.

## 3 Results

### 3.1 Demographic and clinical characteristics

**Figure 1:**
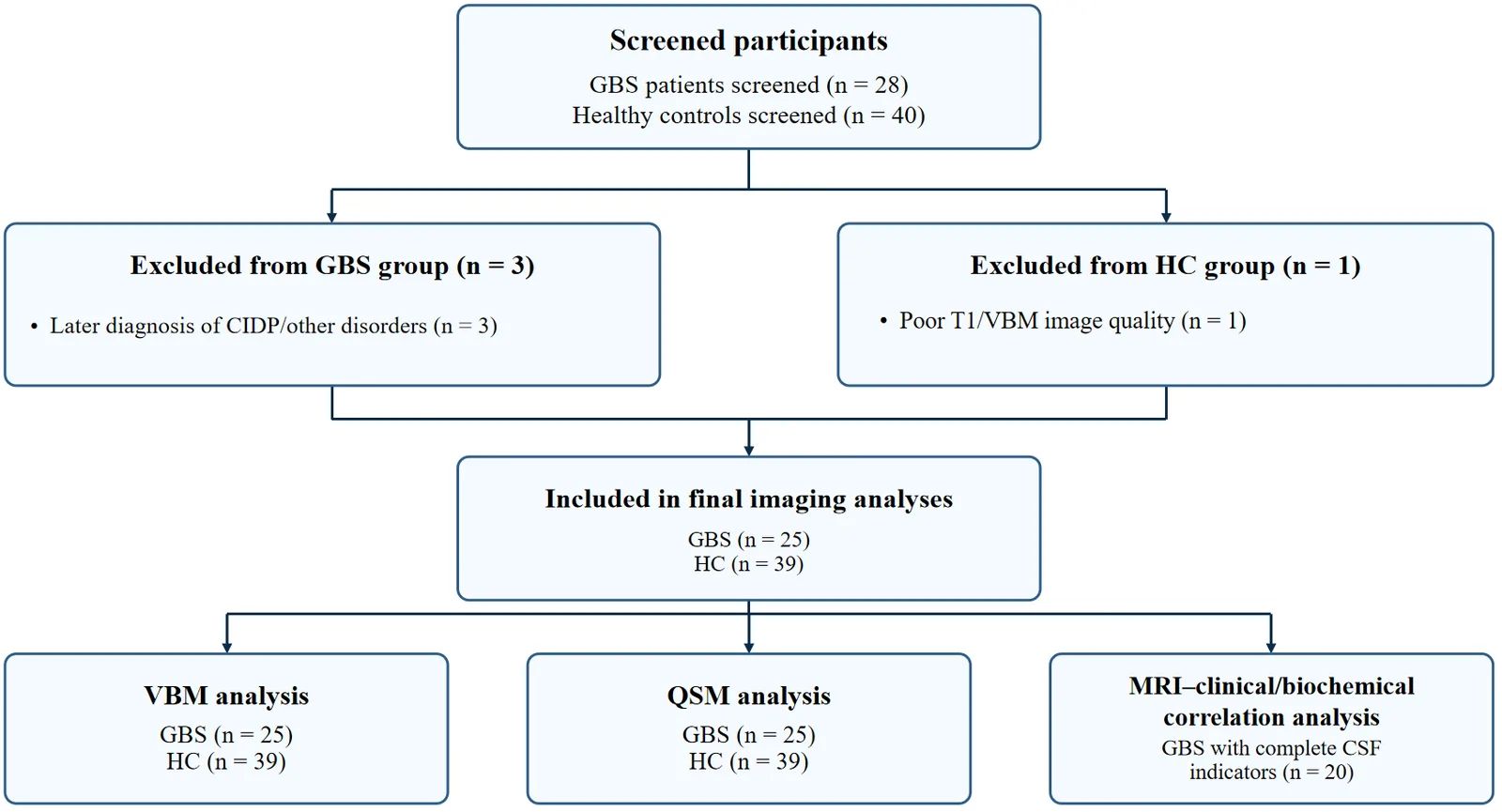
Study flowchart.

**Figure 2:**
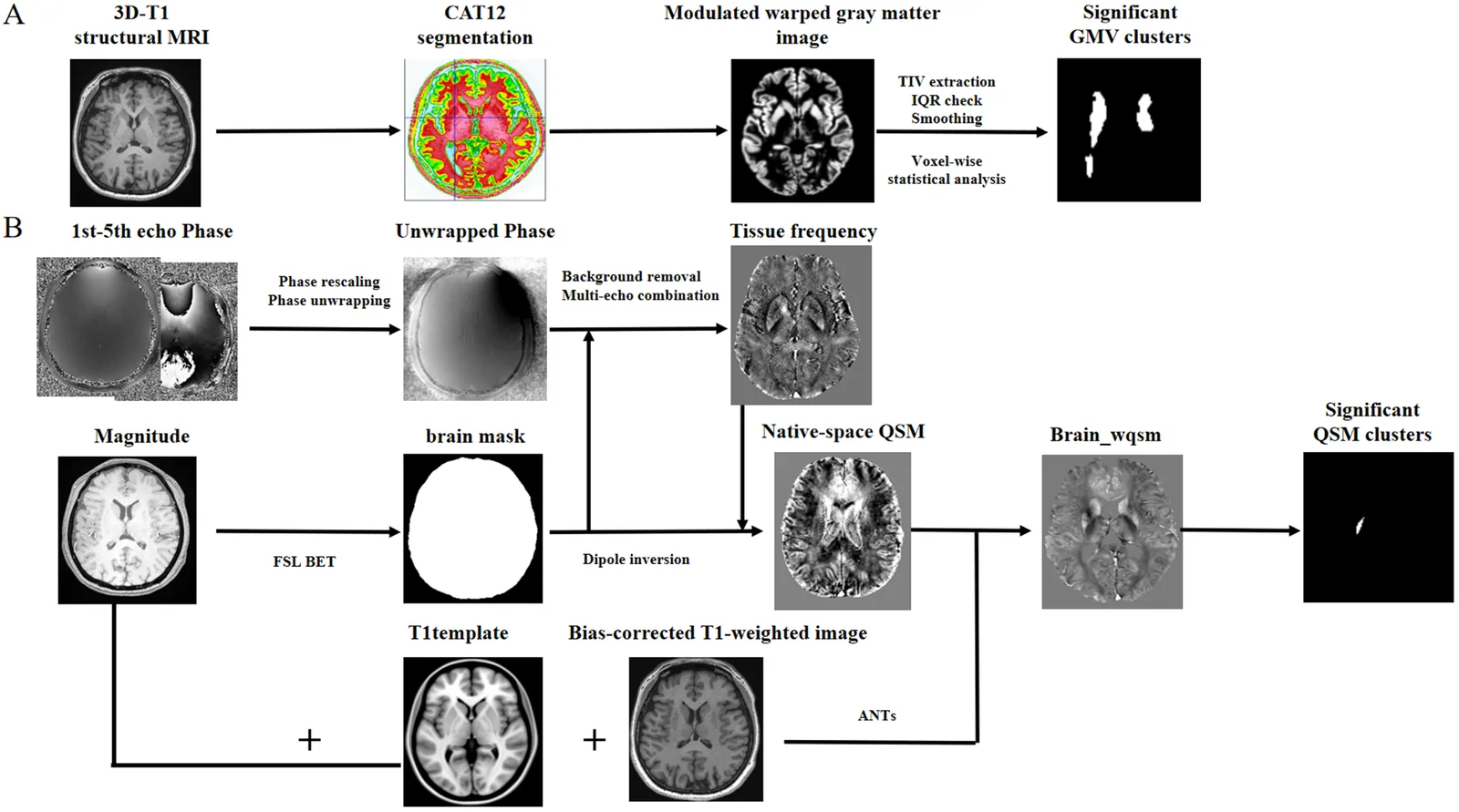
VBM and QSM processing pipelines. **(A)** VBM processing pipeline. **(A)** BSM processing pipeline. VBM, voxel-based morphometry; QSM, quantitative susceptibility mapping.

A total of 28 patients with suspected GBS and 40 healthy controls were initially screened. Three patients were excluded from the GBS group because they were subsequently diagnosed with chronic inflammatory demyelinating polyradiculoneuropathy or other disorders (n = 3). One healthy control was excluded because of poor T1-weighted image quality unsuitable for voxel-based morphometry analysis. Finally, 25 patients with GBS and 39 healthy controls were included in the final imaging analyses, which was close to the estimated sample size requirement, as detailed in **Figure 1**. Because of the limited sample size, patients were grouped as AIDP and other GBS types for descriptive analysis. MRI–clinical/biochemical correlation analyses were performed in 20 patients who had complete cerebrospinal fluid and blood laboratory indicators. There were no significant between-group differences in age, sex distribution, or education years. All patients received intravenous immunoglobulin treatment, except for one patient who received corticosteroid treatment because of financial constraints. Detailed demographic and clinical characteristics are shown in **Table 1**. The detailed image processing workflow is shown in **Figure 2**.

### 3.2 VBM-derived gray matter volume differences

**Figure 3:**
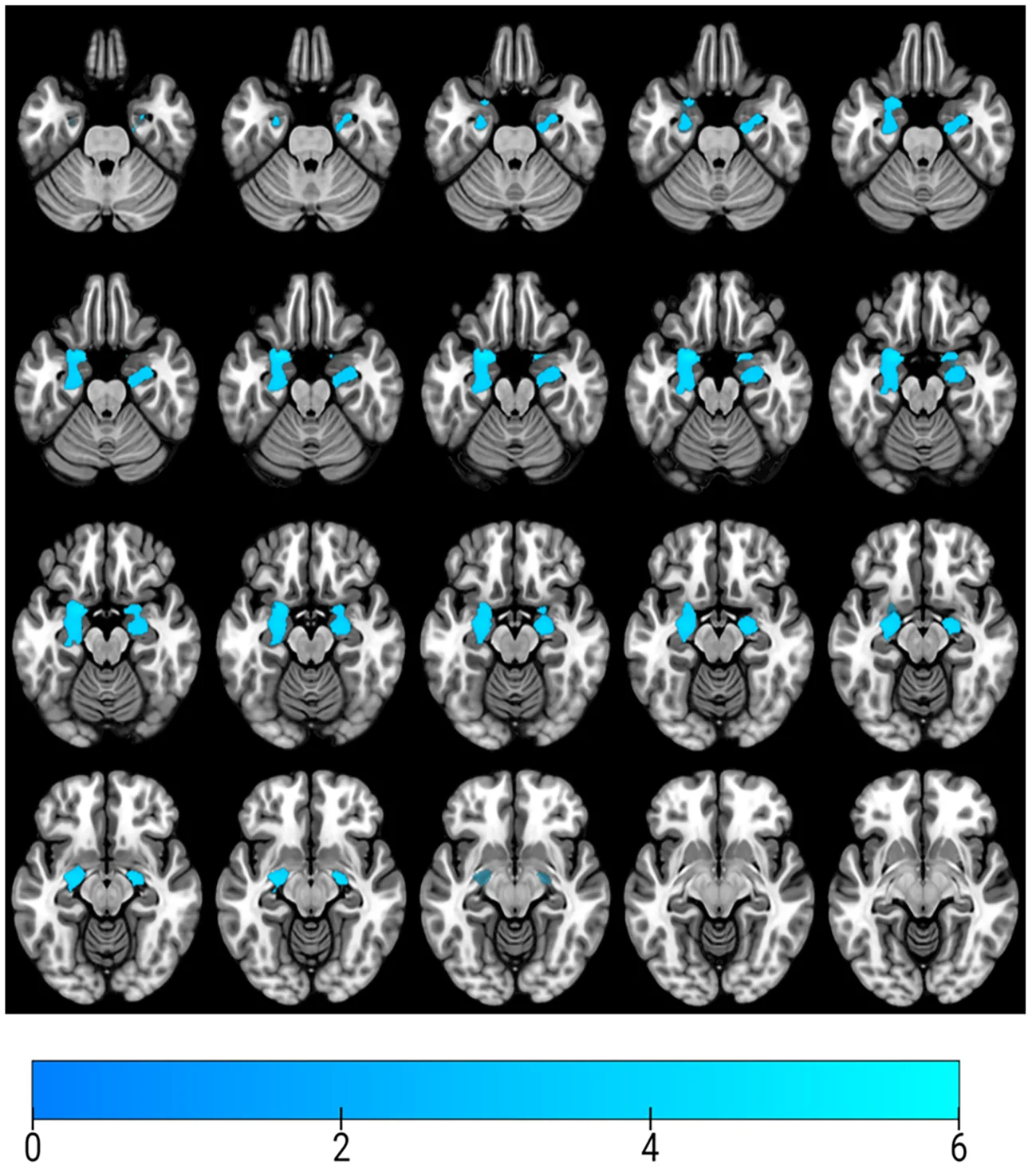
VBM-derived gray matter volume reductions in GBS.

GBS showed significantly reduced GMV in two clusters in the VBM analysis. Cluster 1 was mainly located in the left hippocampus/parahippocampal gyrus/amygdala region and extended to the superior temporal pole, olfactory cortex, orbital part of the inferior frontal gyrus, and insula according to the Automated Anatomical Labeling (AAL) atlas (cluster size = 1324 voxels; peak MNI coordinates: −22.5, 4.5, −21; peak T = 5.3949). Cluster 2 was primarily confined to the right hippocampus/parahippocampal gyrus/amygdala region (cluster size = 825 voxels; peak MNI coordinates: 21, −9, −13.5; peak T = 4.4728). The VBM findings are summarized in **Table 2** and illustrated in **Figure 3**.

**Table 2:** Brain regions with reduced gray matter volume in GBS patients.

Cluster	Brain region (AAL)	Hemisphere	Cluster size (voxels)	Peak MNI coordinates (x, y, z)	Peak T value
1	Hippocampus_L/Amygdala_L/ParaHippocampal_L/Temporal_Pole_Sup_L/Olfactory_L/Frontal_Inf_Orb_L/Insula_L	Left	1324	-22.5, 4.5, -21	5.3949
2	Hippocampus_R/ParaHippocampal_R/Amygdala_R	Right	825	21, -9, -13.5	4.4728

### 3.3 QSM-derived magnetic susceptibility differences

**Figure 4:**
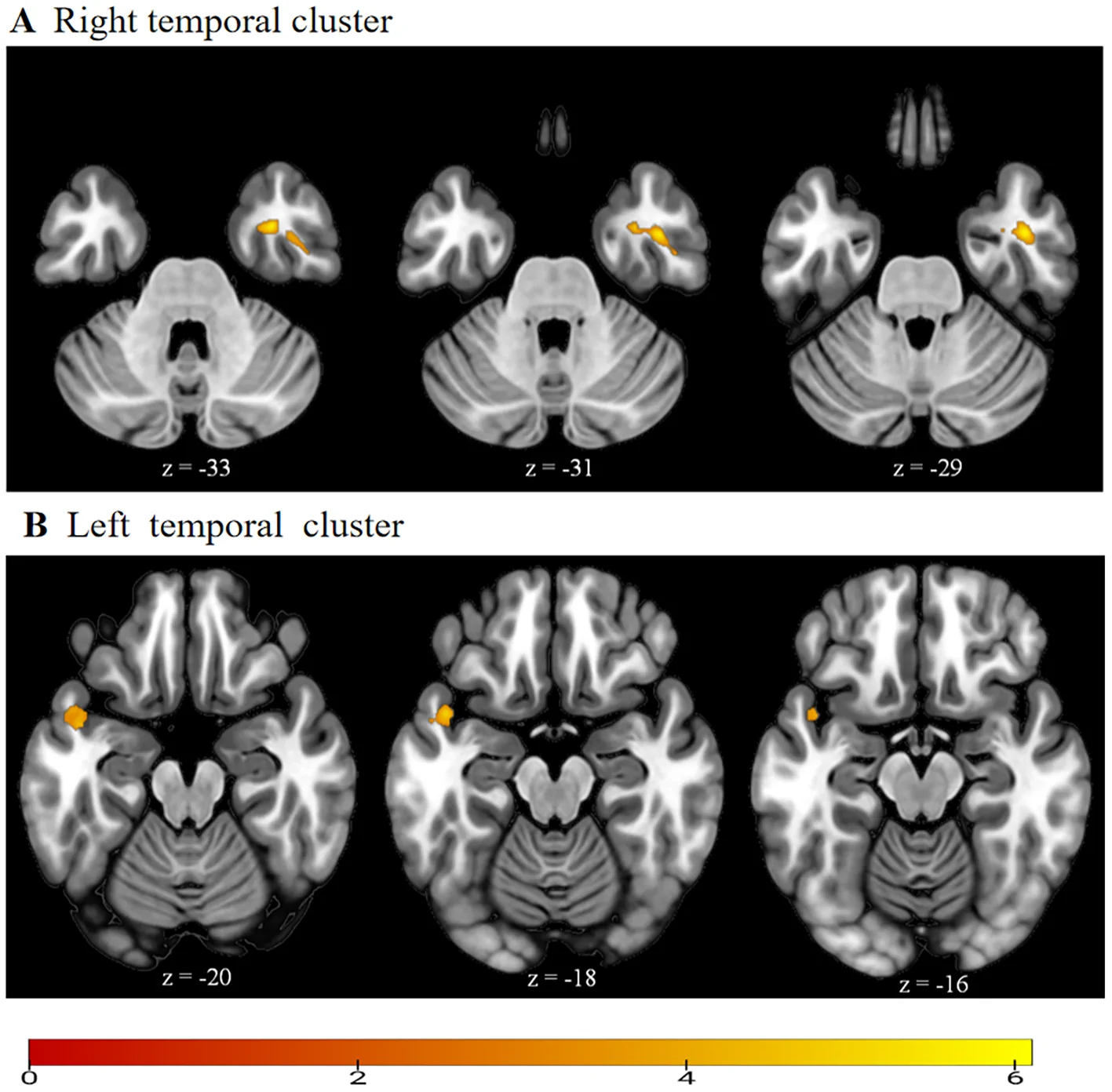
Increased QSM values in patients with GBS. **(A)** Right temporal cluster. **(B)** Left temporal cluster. The color bar represents T values. QSM, quantitative susceptibility mapping; GBS, Guillain -Barré syndrome.

QSM analysis revealed two clusters with significant regional magnetic susceptibility alterations in GBS patients. The GBS group showed increased magnetic susceptibility in the left superior temporal pole and left middle temporal gyrus (cluster size = 356 voxels; peak MNI coordinates: −45, 7, −18; peak T = 5.0624), as well as in the right fusiform gyrus, inferior temporal gyrus, middle temporal gyrus, parahippocampal gyrus, and middle temporal pole (cluster size = 675 voxels; peak MNI coordinates: 44, −3, −30; peak T = 6.0822). The QSM findings are summarized in **Table 3** and illustrated in **Figure 4**.

**Table 3:** Brain regions with increased magnetic susceptibility in GBS patients.

Cluster	Brain region (AAL)	Hemisphere	Cluster size (voxels)	Peak MNI coordinates (x, y, z)	Peak T value
1	Temporal_Pole_Sup_L/Temporal_Mid_L	Left	356	-45, 7, -18	5.0624
2	Fusiform_R/Temporal_Inf_R/Temporal_Mid_R/ParaHippocampal_R/Temporal_Pole_Mid_R	Right	675	44, -3, -30	6.0822

### 3.4 Sensitivity analyses

After diabetes mellitus and hypertension were additionally included as covariates, GBS showed significantly reduced gray matter volume in two clusters. Cluster 1 involved the left hippocampus, amygdala, parahippocampal gyrus, superior temporal pole, olfactory cortex, insula, orbital part of the inferior frontal gyrus, and fusiform gyrus (cluster size = 1543 voxels; peak MNI coordinates: −21, 3, −19.5; peak T = 5.7338). Cluster 2 involved the right hippocampus, parahippocampal gyrus, and amygdala (cluster size = 825 voxels; peak MNI coordinates: 21, −9, −13.5; peak T = 4.3383). For QSM analysis, GBS showed increased magnetic susceptibility in three clusters. Cluster 1 encompassed the left superior temporal pole and middle temporal gyrus (cluster size = 328 voxels; peak MNI coordinates: −45, 7, −18; peak T = 5.0356). Cluster 2 extended across the right fusiform gyrus, middle temporal pole, parahippocampal gyrus, and inferior temporal gyrus (cluster size = 296 voxels; peak MNI coordinates: 35, 0, −33; peak T = 5.8210). Cluster 3 involved the right inferior and middle temporal gyri (cluster size = 347 voxels; peak MNI coordinates: 44, −3, −29; peak T = 6.0485).

Furthermore, after excluding GBS patients with diabetes mellitus, the GBS group had significantly reduced GMV in one cluster involving the left amygdala, hippocampus, superior temporal pole, parahippocampal gyrus, olfactory cortex, orbital part of the inferior frontal gyrus, and insula region (cluster size = 803 voxels; peak MNI coordinates: −21, 3, −21; peak T = 5.7937). For QSM analysis, the GBS group showed increased magnetic susceptibility in one right temporal cluster comprising the right fusiform gyrus, inferior temporal gyrus, middle temporal gyrus, parahippocampal gyrus, and middle temporal pole region (cluster size = 503 voxels; peak MNI coordinates: 43, −3, −30; peak T = 5.2839). In contrast, the GBS group showed decreased magnetic susceptibility in the left putamen and insula region (cluster size = 352 voxels; peak MNI coordinates: −30, 3, 9; peak T = 4.6594). Full results of these sensitivity analyses are presented in **Supplementary Tables S1** and **S2**.

### 3.5 Exploratory associations between imaging-derived ROIs and biofluid markers

**Figure 5:**
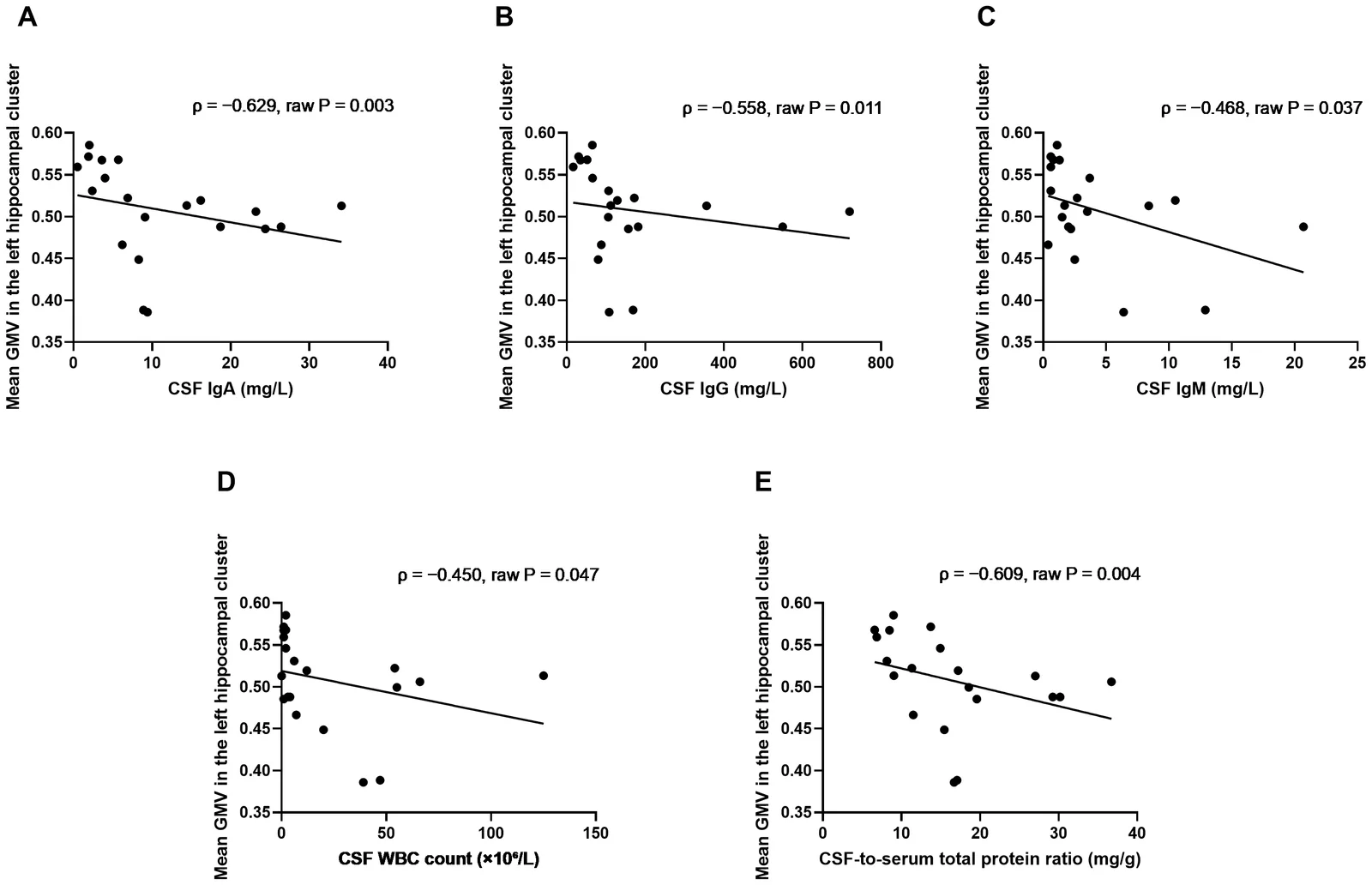
Exploratory associations between imaging-derived ROIs and CSF markers. Associations of mean GMV in the left hippocampal cluster with **(A)** CSF IgA, **(B)** CSF IgG, **(C)** CSF IgM, **(D)** CSF WBC count, and **(E)** the CSF-to-serum total protein ratio. GMV, gray matter volume; ROI, region of interest; CSF, cerebrospinal fluid; WBC, white blood cell.

Spearman rank-correlation analyses revealed that GMV in Cluster 1, corresponding to the left hippocampal cluster, was negatively correlated with CSF IgA (ρ = −0.629, raw P = 0.003), CSF IgG (ρ = −0.558, raw P = 0.011), CSF IgM (ρ = −0.468, raw P = 0.037), CSF white blood cell count (ρ = −0.450, raw P = 0.047), and the CSF-to-serum total protein ratio (ρ = −0.609, raw P = 0.004). After Benjamini–Hochberg FDR correction the negative correlations of Cluster 1 GMV with CSF IgA (FDR-adjusted ρ = 0.026), CSF IgG (FDR-adjusted ρ = 0.042), and the CSF-to-serum total protein ratio (FDR-adjusted ρ = 0.026) remained statistically significant (**Table 4**, **Figure 5**). After excluding the four patients with CSF WBC counts >50 × 10^6^/L, the negative association between GMV in the left hippocampal cluster and CSF WBC count persisted (ρ = −0.664, raw P = 0.005, q = 0.016). Full results of the sensitivity analysis are presented in **Supplementary Table S3** and **Supplementary Figures S1** and **S2**. No significant correlations were observed between QSM values in the significant clusters and CSF indicators. No significant correlations were found between blood indicators and GMV values in the significant VBM clusters or QSM values in the significant QSM clusters.

**Table 4:** Exploratory correlations between VBM-ROI gray matter volume and CSF markers in GBS.

ROIs	CSF markers	Spearman ρ	Raw P	FDR-adjusted q
Cluster 1	CSF IgA	-0.629	0.003^*^	0.026^*^
Cluster 1	CSF IgG	-0.558	0.011^*^	0.042^*^
Cluster 1	CSF IgM	-0.468	0.037^*^	0.112
Cluster 1	CSF-WBC	-0.450	0.047^*^	0.112
Cluster 1	CSF-to-blood glucose ratio	0.251	0.286	0.286
Cluster 1	CSF-to-serum total protein ratio	-0.609	0.004^*^	0.026^*^
Cluster 2	CSF IgA	-0.286	0.222	0.242
Cluster 2	CSF IgG	-0.293	0.210	0.242
Cluster 2	CSF IgM	-0.309	0.185	0.242
Cluster 2	CSF-WBC	-0.407	0.075	0.150
Cluster 2	CSF-to-blood glucose ratio	0.286	0.222	0.242
Cluster 2	CSF-to-serum total protein ratio	-0.304	0.193	0.242

Exploratory correlation analyses were performed using Spearman rank correlation in 20 patients with GBS who had available cerebrospinal fluid data. Cluster 1 corresponds to the left hippocampal/amygdala/parahippocampal cluster, and Cluster 2 corresponds to the right hippocampal/parahippocampal/amygdala cluster. Raw P values < 0.05 are marked with an asterisk (*). Multiple comparisons were corrected using the Benjamini–Hochberg false discovery rate (FDR) procedure, with q < 0.05 considered statistically significant.

## 4 Discussion

In the present study, we used VBM and QSM to investigate brain gray matter volume and susceptibility-related tissue alterations in patients with GBS. The main findings were that GBS patients showed reduced GMV in the bilateral hippocampal–parahippocampal–amygdala regions, together with increased magnetic susceptibility in bilateral temporal regions. Exploratory ROI-based analyses further revealed associations between hippocampal/parahippocampal gray matter volume and multiple CSF markers. These findings suggest that GBS, although classically regarded as an acute immune-mediated peripheral neuropathy, may be accompanied by subtle but measurable alterations in central brain structure and susceptibility-related tissue properties. Importantly, the exploratory associations between limbic gray matter alterations and CSF metabolic or immune-inflammatory indicators provide preliminary evidence that GBS-related brain changes may be linked to disease-related biological processes rather than representing isolated imaging findings.

The most prominent structural finding of the present study was reduced GMV in the bilateral hippocampal–parahippocampal–amygdala regions in patients with GBS, with more extensive abnormalities on the left side extending to the superior temporal pole, olfactory cortex, orbital part of the inferior frontal gyrus, and insula. The hippocampus, parahippocampal gyrus, and amygdala are key components of the medial temporal–limbic system and are jointly involved in contextual memory, emotional and stress responses, and modulation of the affective dimension of pain (34, 35). Previous studies have demonstrated that the hippocampus and amygdala exhibit substantial structural plasticity and may be particularly vulnerable to chronic stress and inflammation-related processes (36). In GBS, immune-mediated injury to peripheral nerves and nerve roots may substantially disrupt sensory and motor afferent–efferent signaling, while patients frequently experience limb weakness, sensory disturbances, pain, and disease-related stress (5, 37). Previous studies of trigeminal neuralgia, postherpetic neuralgia, cervical spondylotic myelopathy, and other peripheral or spinal nervous system disorders have shown that neuropathic pain, aberrant sensory input, or dysfunction of spinal pathways may be accompanied by plastic reorganization of central sensorimotor networks (38–42). Although these disorders differ from GBS in terms of disease course and underlying pathophysiology, these findings provide indirect support for the possibility that GBS may also be accompanied by secondary structural remodeling of the brain. On the basis of these findings, GBS-related peripheral neuroimmune injury, altered sensorimotor input, pain, and disease-related stress may all be considered potential contributors to the imaging abnormalities observed in the present study.

The present study revealed increased magnetic susceptibility in bilateral temporal regions in patients with GBS. The left-sided abnormality predominantly involved the superior temporal pole and middle temporal gyrus, whereas the right-sided abnormality was more extensive, encompassing the fusiform gyrus, inferior temporal gyrus, middle temporal gyrus, parahippocampal gyrus, and middle temporal pole. These regions spanning the anterolateral, ventral, and medial temporal cortices are involved in multimodal information integration, semantic processing, socioemotional processing, higher-order visual recognition, contextual representation, and memory processing (43, 44). These findings therefore suggest that GBS-related brain alterations may not be confined to the medial temporal–limbic system observed in the aforementioned VBM analysis, but may also extend to the anterior temporal cortex and higher-order temporal association areas. Increased QSM signal generally indicates an increase in local paramagnetic substances, of which ferritin stored in glial cells is the major source (45). Previous autopsy studies have observed inflammatory cell infiltration, microglial activation, and secondary myelin changes in central nervous system regions such as the spinal cord and brainstem in some patients with GBS (10); meanwhile, other clinical studies have reported elevated CSF levels of cytokines including IL-17 and IL-22 (46, 47). These lines of evidence suggest that a subset of GBS patients may exhibit central nervous system inflammatory responses. Inflammatory stimuli can affect cellular iron uptake, storage, and export by regulating the expression of iron metabolism-related proteins such as DMT1, FPN1, and hepcidin, thereby disrupting local iron homeostasis (21, 48). In addition, animal studies have suggested that persistent pain may also influence iron metabolism in similar brain regions (49). Therefore, the increased temporal lobe magnetic susceptibility observed in the present study may reflect a local iron homeostasis imbalance driven by the combined effects of GBS-related immune inflammation, pain, and other disease-related stress factors.

From a clinical perspective, reduced GMV in the hippocampal–parahippocampal–amygdala regions and increased magnetic susceptibility in the temporal regions raise the possibility that, in addition to typical sensorimotor deficits, GBS may be accompanied by cognitive or emotional alterations. Previous clinical studies have shown that GBS patients may experience prominent anxiety and sleep disturbances during the acute phase, while depression, sleep abnormalities, and persistent fatigue may continue into the recovery phase (50–52). These observations highlight the need to determine whether hippocampal–amygdala structural alterations and temporal lobe magnetic susceptibility alterations are associated with subsequent memory and emotional outcomes in patients with GBS. However, because standardized assessments of pain severity, emotional symptoms, cognitive function, and sensory abnormalities were not administered, the present study cannot establish whether the observed GMV reductions and magnetic susceptibility increases were accompanied by measurable memory or emotional dysfunction. Furthermore, given the cross-sectional design, it remains unclear whether these alterations represent acute disease-related effects, chronic structural injury, transient changes in tissue status, or pre-existing interindividual differences. These interpretations therefore remain hypothetical and require validation in longitudinal imaging studies incorporating standardized clinical assessments.

The sensitivity analyses further supported the relative robustness of the principal neuroimaging findings. After diabetes mellitus and hypertension were additionally included as covariates, the overall spatial distributions of reduced GMV in the bilateral hippocampal–parahippocampal–amygdala regions and increased magnetic susceptibility in the bilateral temporal regions were largely preserved, suggesting that these abnormalities were unlikely to be fully explained by common vascular and metabolic comorbidities. After patients with GBS and comorbid diabetes mellitus were further excluded, reduced GMV in the left hippocampal–parahippocampal–amygdala region and increased magnetic susceptibility in the right temporal region remained significant, indicating that these two alterations may represent relatively more robust neuroimaging findings in the present study. In contrast, reduced GMV in the right hippocampal-related cluster and increased magnetic susceptibility in the left temporal region no longer reached statistical significance after the exclusion of patients with diabetes mellitus. This may indicate that the effects in the right hippocampal and left temporal regions were relatively weaker and the reduction in sample size following patient exclusion may have decreased statistical power.

Exploratory correlation analyses showed that GMV in the left hippocampal–parahippocampal–amygdala cluster was negatively associated with CSF IgA, IgG, IgM, CSF white blood cell count, and the CSF-to-serum total protein ratio. These associations may involve several processes, including GBS-related systemic immune activation, alterations in neural barrier function, and disturbances in CSF dynamics, and may provide indirect evidence of the potential immune vulnerability of the medial temporal–limbic system. Studies in community-based and generally healthy populations have linked higher peripheral levels of IL-6, CRP, and other inflammatory markers to lower hippocampal or parahippocampal gray matter volume (17, 19, 53, 54). Structural MRI studies have further reported reduced hippocampal or amygdalar volumes in systemic autoimmune or inflammatory diseases, including rheumatoid arthritis, systemic lupus erythematosus, and ulcerative colitis (55–57). Although the pathophysiological mechanisms of these conditions differ from those of GBS and cannot be directly extrapolated to the present findings, collectively, these observations support the potential immune vulnerability of the medial temporal–limbic system. Previous studies have shown that GBS may be accompanied by increased permeability of the blood–nerve barrier (BNB) and the blood–spinal nerve root barrier (58–60). In parallel, elevated CSF total protein and an abnormal cerebrospinal fluid-to-serum albumin quotient (QAlb) are commonly observed in GBS and may partly reflect alterations in BCSFB function or CSF dynamics (61–63); however, these findings do not directly demonstrate disruption of the brain parenchymal blood–brain barrier (BBB) or barrier disruption within hippocampal tissue. Paired CSF–serum oligoclonal band studies further indicate that IgG abnormalities in GBS are often characterized by matched or “mirror-pattern” bands in serum and CSF, supporting systemic immune activation and the transfer of blood-derived immunoglobulins, whereas true intrathecal immunoglobulin synthesis appears to be relatively uncommon (64–67). Because CSF albumin measurements were unavailable for some participants, we were unable to reliably calculate QAlb or the immunoglobulin G index (IgG index). Therefore, the present associations cannot determine whether the immunoglobulin-related findings were driven primarily by barrier-related entry of blood-derived immunoglobulins or by true intrathecal immunoglobulin synthesis, nor do they directly demonstrate brain parenchymal BBB disruption.

Notably, although GMV reductions were observed in bilateral hippocampal–parahippocampal–amygdala regions, significant associations with multiple CSF immune- and protein-related markers were detected only in the left-sided cluster, suggesting that the link between CSF inflammatory burden and medial temporal–limbic structural vulnerability may be more pronounced in the left hemisphere. Prior studies have indicated that brain–immune interactions may exhibit some degree of hemispheric asymmetry (68); similarly, Marsland et al. reported a more stable statistical association between peripheral IL-6 levels and hippocampal structure in the left hemisphere (17). No significant correlations were observed between QSM values and CSF markers. This may be because QSM is influenced by multiple tissue properties, including iron content, myelin, tissue water, and venous oxygenation, whereas routine CSF markers do not directly capture these local microstructural changes (69). In addition, no significant associations were found between routine blood-based markers and the significant imaging measures, which may be attributable to the susceptibility of peripheral blood indices to various physiological and clinical factors, as well as the limited range of immune markers examined in the present study.

However, the present study still has several limitations. First, the single-center design and limited sample size may potentially reduce statistical power; the cross-sectional design precluded determination of the temporal dynamics and causal basis of the observed neuroimaging alterations. Second, due to the exclusion of patients with extremely unstable clinical conditions, severe respiratory muscle involvement, or impaired consciousness, there might be a selection bias in this study. Third, because HCs did not undergo lumbar puncture, CSF data were unavailable for this group; therefore, imaging–CSF correlation analyses were restricted to patients with GBS. Fourth, QSM is sensitive to multiple tissue components and cannot distinguish the underlying pathological substrates. Moreover, direct markers of iron homeostasis, such as ferritin, transferrin, hepcidin, and soluble transferrin receptor, were not measured in either blood or CSF. Therefore, the observed susceptibility alterations cannot be definitively attributed to disturbed iron metabolism. Therefore, future studies will include larger multicenter cohorts, conduct longitudinal MRI follow-up, and combine paired blood and cerebrospinal fluid inflammatory factors, iron metabolism indicators, oxidative stress indicators, and more comprehensive clinical phenotypic assessments, in order to further validate these imaging findings and clarify the potential mechanisms underlying GBS-related brain alterations.

## 5 Conclusion

Overall, the results of the present study suggest that GBS, as an acute peripheral immune-mediated disease, may not be completely confined to the peripheral nervous system. Its related immune-inflammatory processes may influence central tissue status. By combining VBM and QSM, the present study identified structural and magnetic susceptibility alterations in multiple brain regions, which were significantly associated with CSF markers, providing preliminary neuroimaging evidence from both the macroscopic gray matter structural level and the microscopic tissue magnetic susceptibility level and offering new research clues for further understanding central–peripheral interactions in GBS.

## Data Availability

The raw data supporting the conclusions of this article can be made available by the authors on reasonable request.

## References

[1] Shahrizaila N Lehmann HC Kuwabara S . Guillain-barré syndrome. Lancet. (2021) 397:1214–28. doi: 10.1016/S0140-6736(21)00517-1 33647239

[2] Willison HJ Jacobs BC Van Doorn PA . Guillain-barré syndrome. Lancet. (2016) 388:717–27. doi: 10.1016/S0140-6736(16)00339-1 26948435

[3] Gào X Zhao C Yang J Yang Z Feng J Zhan S . Impact of COVID-19 vaccination coverage on global disability burden of Guillain-Barré syndrome. NPJ Vaccines. (2025) 10:182. doi: 10.1038/s41541-025-01239-1 40753095 PMC12318086

[4] van den Berg B Storm EF Garssen MJP Blomkwist-Markens PH Jacobs BC . Clinical outcome of Guillain-Barré syndrome after prolonged mechanical ventilation. J Neurol Neurosurg Psychiatry. (2018) 89:949–54. doi: 10.1136/jnnp-2018-317968 29627773

[5] Leonhard SE Mandarakas MR Gondim FAA Bateman K Ferreira MLB Cornblath DR . Diagnosis and management of Guillain-Barré syndrome in ten steps. Nat Rev Neurol. (2019) 15:671–83. doi: 10.1038/s41582-019-0250-9 31541214 PMC6821638

[6] Mulroy E Anderson NE . Altered mental status in “Guillain-Barré syndrome” -a noteworthy clinical clue. Ann Clin Transl Neurol. (2020) 7:2489–507. doi: 10.1002/acn3.51226 33136342 PMC7732251

[7] Doxaki C Papadopoulou E Maniadaki I Tsakalis NG Palikaras K Vorgia P . Case report: intracranial hypertension secondary to guillain-barre syndrome. Front Pediatr. (2020) 8:608695. doi: 10.3389/fped.2020.608695 33553071 PMC7857149

[8] Chen A Kim J Henderson G Berkowitz A . Posterior reversible encephalopathy syndrome in Guillain-Barré syndrome. J Clin Neurosci. (2015) 22:914–6. doi: 10.1016/j.jocn.2014.11.004 25800144

[9] Zhao PP Ji QK Sui RB Zhang R Zhang LJ Xu ZX . Increased intracranial pressure in Guillain-Barré syndrome: A case report. Med (Baltimore). (2018) 97:e11584. doi: 10.1097/MD.0000000000011584 30045288 PMC6078745

[10] Maier H Schmidbauer M Pfausler B Schmutzhard E Budka H . Central nervous system pathology in patients with the Guillain-Barré syndrome. Brain. (1997) 120:451–64. doi: 10.1093/brain/120.3.451 9126057

[11] Taylor KS Anastakis DJ Davis KD . Cutting your nerve changes your brain. Brain. (2009) 132:3122–33. doi: 10.1093/brain/awp231 19737843

[12] Meyers EC Kasliwal N Solorzano BR Lai E Bendale G Berry A . Enhancing plasticity in central networks improves motor and sensory recovery after nerve damage. Nat Commun. (2019) 10:5782. doi: 10.1038/s41467-019-13695-0 31857587 PMC6923364

[13] Li N Downey JE Bar-Shir A Gilad AA Walczak P Kim H . Optogenetic-guided cortical plasticity after nerve injury. Proc Natl Acad Sci USA. (2011) 108:8838–43. doi: 10.1073/pnas.1100815108 21555573 PMC3102379

[14] Li C Liu SY Pi W Zhang PX . Cortical plasticity and nerve regeneration after peripheral nerve injury. Neural Regener Res. (2021) 16:1518–23. doi: 10.4103/1673-5374.303008 33433465 PMC8323687

[15] Björkman A Weibull A . Loss of inhibition in ipsilateral somatosensory areas following altered afferent nerve signaling from the hand. Neurosci Res. (2018) 135:32–6. doi: 10.1016/j.neures.2017.12.004 29258852

[16] van Doorn PA Van den Bergh PYK Hadden RDM Avau B Vankrunkelsven P Attarian S . European Academy of Neurology/Peripheral Nerve Society Guideline on diagnosis and treatment of Guillain-Barré syndrome. Eur J Neurol. (2023) 30:3646–74. doi: 10.1111/ene.16073 37814552

[17] Marsland AL Gianaros PJ Abramowitch SM Manuck SB Hariri AR . Interleukin-6 covaries inversely with hippocampal grey matter volume in middle-aged adults. Biol Psychiatry. (2008) 64:484–90. doi: 10.1016/j.biopsych.2008.04.016 18514163 PMC2562462

[18] Marsland AL Gianaros PJ Kuan DCH Sheu LK Krajina K Manuck SB . Brain morphology links systemic inflammation to cognitive function in midlife adults. Brain Behav Immun. (2015) 48:195–204. doi: 10.1016/j.bbi.2015.03.015 25882911 PMC4508197

[19] Satizabal CL Zhu YC Mazoyer B Dufouil C Tzourio C . Circulating IL-6 and CRP are associated with MRI findings in the elderly: the 3C-Dijon Study. Neurology. (2012) 78:720–7. doi: 10.1212/WNL.0b013e318248e50f 22357713

[20] Liddelow SA Guttenplan KA Clarke LE Bennett FC Bohlen CJ Schirmer L . Neurotoxic reactive astrocytes are induced by activated microglia. Nature. (2017) 541:481–7. doi: 10.1038/nature21029 28099414 PMC5404890

[21] Urrutia P Aguirre P Esparza A Tapia V Mena NP Arredondo M . Inflammation alters the expression of DMT1, FPN1 and hepcidin, and it causes iron accumulation in central nervous system cells. J Neurochem. (2013) 126:541–9. doi: 10.1111/jnc.12244 23506423

[22] Sun T Chen X Shi S Liu Q Cheng Y . Peripheral blood and cerebrospinal fluid cytokine levels in guillain barré Syndrome: A systematic review and meta-analysis. Front Neurosci. (2019) 13:717. doi: 10.3389/fnins.2019.00717 31379477 PMC6646663

[23] Farokhian F Beheshti I Sone D Matsuda H . Comparing CAT12 and VBM8 for detecting brain morphological abnormalities in temporal lobe epilepsy. Front Neurol. (2017) 8:428. doi: 10.3389/fneur.2017.00428 28883807 PMC5573734

[24] Harada T Kudo K Fujima N Yoshikawa M Ikebe Y Sato R . Quantitative susceptibility mapping: basic methods and clinical applications. Radiographics. (2022) 42:1161–76. doi: 10.1148/rg.210054 35522577

[25] 中华医学会神经病学分会 . 中国吉兰-巴雷综合征诊治指南2024. 中华神经科杂志. (2024) 57:936. doi: 10.3760/cma.j.cn113694-20240220-00099 30704229

[26] Kang H . Sample size determination and power analysis using the G*Power software. J Educ Eval Health Prof. (2021) 18:17. doi: 10.3352/jeehp.2021.18.17 34325496 PMC8441096

[27] Gaser C Dahnke R Thompson PM Kurth F Luders E The Alzheimer’s Disease Neuroimaging Initiative null . CAT: a computational anatomy toolbox for the analysis of structural MRI data. GigaScience. (2024) 13:giae049. doi: 10.1093/gigascience/giae049 39102518 PMC11299546

[28] Dahnke R Kalc P Ziegler G Grosskreutz J Gaser C . Segmentation-based quality control of structural MRI using the CAT12 toolbox. GigaScience. (2025) 14:giaf146. doi: 10.1093/gigascience/giaf146 41316989 PMC12758382

[29] Malone IB Leung KK Clegg S Barnes J Whitwell JL Ashburner J . Accurate automatic estimation of total intracranial volume: a nuisance variable with less nuisance. Neuroimage. (2015) 104:366–72. doi: 10.1016/j.neuroimage.2014.09.034 25255942 PMC4265726

[30] Kurth F Gaser C Luders E . A 12-step user guide for analyzing voxel-wise gray matter asymmetries in statistical parametric mapping (SPM). Nat Protoc. (2015) 10:293–304. doi: 10.1038/nprot.2015.014 25591011

[31] Chen L Soldan A Faria A Albert M van Zijl PCM Li X . Susceptibility MRI helps predict mild cognitive impairment onset and cognitive decline in cognitively unimpaired older adults. Radiology. (2025) 316:e250513. doi: 10.1148/radiol.250513 40923890 PMC12501624

[32] QSM Consensus Organization Committee Bilgic B Costagli M Chan KS Duyn J Langkammer C . Recommended implementation of quantitative susceptibility mapping for clinical research in the brain: A consensus of the ISMRM electro‐magnetic tissue properties study group. Magn Reson Med. (2024) 91:1834–62. doi: 10.1002/mrm.30006 38247051 PMC10950544

[33] Wang X Xiong Y Liu X Zong X Duan C Wu Y . Subcortical nuclei and glymphatic system alterations in migraine with aura: a comparative 7T multimodal MRI analysis. J Headache Pain. (2025) 27:22. doi: 10.1186/s10194-025-02258-y 41462078 PMC12825203

[34] Dahlgren K Ferris C Hamann S . Neural correlates of successful emotional episodic encoding and retrieval: An SDM meta-analysis of neuroimaging studies. Neuropsychologia. (2020) 143:107495. doi: 10.1016/j.neuropsychologia.2020.107495 32416099

[35] McEwen BS Nasca C Gray JD . Stress effects on neuronal structure: hippocampus, amygdala, and prefrontal cortex. Neuropsychopharmacology. (2016) 41:3–23. doi: 10.1038/npp.2015.171 26076834 PMC4677120

[36] Leuner B Shors TJ . Stress, anxiety, and dendritic spines: what are the connections? Neuroscience. (2013) 251:108–19. doi: 10.1016/j.neuroscience.2012.04.021 22522470

[37] Ruts L Drenthen J Jongen JLM Hop WCJ Visser GH Jacobs BC . Pain in Guillain-Barre syndrome: a long-term follow-up study. Neurology. (2010) 75:1439–47. doi: 10.1212/WNL.0b013e3181f88345 20861454

[38] Obermann M Rodriguez-Raecke R Naegel S Holle D Mueller D Yoon MS . Gray matter volume reduction reflects chronic pain in trigeminal neuralgia. Neuroimage. (2013) 74:352–8. doi: 10.1016/j.neuroimage.2013.02.029 23485849

[39] Liu J Gu L Huang Q Hong S Zeng X Zhang D . Altered gray matter volume in patients with herpes zoster and postherpetic neuralgia. JPR. (2019) 12:605–16. doi: 10.2147/JPR.S183561 30799946 PMC6369852

[40] Zhang Y Cao S Yuan J Song G Yu T Liang X . Functional and structural changes in postherpetic neuralgia brain before and six months after pain relieving. J Pain Res. (2020) 13:909–18. doi: 10.2147/JPR.S246745 32440196 PMC7210030

[41] Wu X Wang Y Chang J Zhu K Zhang S Li Y . Remodeling of the brain correlates with gait instability in cervical spondylotic myelopathy. Front Neurosci. (2023) 17:1087945. doi: 10.3389/fnins.2023.1087945 36816111 PMC9932596

[42] Grabher P Mohammadi S Trachsler A Friedl S David G Sutter R . Voxel-based analysis of grey and white matter degeneration in cervical spondylotic myelopathy. Sci Rep. (2016) 6:24636. doi: 10.1038/srep24636 27095134 PMC4837346

[43] Herlin B Navarro V Dupont S . The temporal pole: From anatomy to function-A literature appraisal. J Chem Neuroanat. (2021) 113:101925. doi: 10.1016/j.jchemneu.2021.101925 33582250

[44] Rice GE Lambon Ralph MA Hoffman P . The roles of left versus right anterior temporal lobes in conceptual knowledge: an ALE meta-analysis of 97 functional neuroimaging studies. Cereb Cortex. (2015) 25:4374–91. doi: 10.1093/cercor/bhv024 25771223 PMC4816787

[45] Langkammer C Schweser F Krebs N Deistung A Goessler W Scheurer E . Quantitative susceptibility mapping (QSM) as a means to measure brain iron? A post mortem validation study. Neuroimage. (2012) 62:1593–9. doi: 10.1016/j.neuroimage.2012.05.049 22634862 PMC3413885

[46] Li S Yu M Li H Zhang H Jiang Y . IL-17 and IL-22 in cerebrospinal fluid and plasma are elevated in Guillain-Barré syndrome. Mediators Inflammation. (2012) 2012:260473. doi: 10.1155/2012/260473 23091305 PMC3468147

[47] Debnath M Nagappa M Dutta D Talukdar PM Subbanna M Shivakumar V . Evidence of altered Th17 pathway signatures in the cerebrospinal fluid of patients with Guillain Barré Syndrome. J Clin Neurosci. (2020) 75:176–80. doi: 10.1016/j.jocn.2020.03.010 32217048

[48] Pandur E Varga E Tamási K Pap R Nagy J Sipos K . Effect of inflammatory mediators lipopolysaccharide and lipoteichoic acid on iron metabolism of differentiated SH-SY5Y cells alters in the presence of BV-2 microglia. Int J Mol Sci. (2018) 20:17. doi: 10.3390/ijms20010017 30577543 PMC6337407

[49] Tang Y Liu C Zhu T Chen H Sun Y Zhang X . Transcriptome profiles of incRNA and mRNA highlight the role of ferroptosis in chronic neuropathic pain with memory impairment. Front Cell Dev Biol. (2022) 10:843297. doi: 10.3389/fcell.2022.843297 35547819 PMC9082550

[50] Levison LS Thomsen RW Andersen H . Increased risk of depression after Guillain-Barré syndrome. Muscle Nerve. (2023) 67:497–505. doi: 10.1002/mus.27816 36906822

[51] Bahnasy WS El-Heneedy YAE El-Shamy AM Badr MY Amer RA Ibrahim ISE . Sleep and psychiatric abnormalities in Gullian Barré syndrome. Egypt J Neurol Psychiatr Neurosurg. (2018) 54:5. doi: 10.1186/s41983-018-0007-1 29780225 PMC5954782

[52] Karkare K Sinha S Taly AB Rao S . Prevalence and profile of sleep disturbances in Guillain-Barre syndrome: a prospective questionnaire-based study during 10 days of hospitalization. Acta Neurol Scand. (2013) 127:116–23. doi: 10.1111/j.1600-0404.2012.01688.x 22642612

[53] Zhuo B Zheng D Cai M Wang C Zhang S Zhang Z . Mediation effect of brain volume on the relationship between peripheral inflammation and cognitive decline. J Alzheimers Dis. (2023) 95:523–33. doi: 10.3233/JAD-230253 37545239

[54] Walker KA Hoogeveen RC Folsom AR Ballantyne CM Knopman DS Windham BG . Midlife systemic inflammatory markers are associated with late-life brain volume: The ARIC study. Neurology. (2017) 89:2262–70. doi: 10.1212/WNL.0000000000004688 29093073 PMC5705246

[55] Cox JG de Groot M Kempton MJ Williams SCR Cole JH . Comparison of volumetric brain analysis in subjects with rheumatoid arthritis and ulcerative colitis. Front Med (Lausanne). (2024) 11:1468910. doi: 10.3389/fmed.2024.1468910 39635596 PMC11614619

[56] Cox JG de Groot M Cole JH Williams SCR Kempton MJ . A meta-analysis of structural MRI studies of the brain in systemic lupus erythematosus (SLE). Clin Rheumatol. (2023) 42:319–26. doi: 10.1007/s10067-022-06482-8 36534349 PMC9873736

[57] Emmer BJ van der Grond J Steup-Beekman GM Huizinga TWJ van Buchem MA . Selective involvement of the amygdala in systemic lupus erythematosus. PLoS Med. (2006) 3:e499. doi: 10.1371/journal.pmed.0030499 17177602 PMC1702559

[58] Kanda T Yamawaki M Mizusawa H . Sera from Guillain-Barré patients enhance leakage in blood-nerve barrier model. Neurology. (2003) 60:301–6. doi: 10.1212/01.wnl.0000041494.70178.17 12552049

[59] Franciotta D Gastaldi M Zardini E Nobile-Orazio E . Cerebrospinal fluid total protein determination in acute and chronic inflammatory demyelinating polyneuropathies: a critical reappraisal. J Peripher Nerv Syst. (2018) 23:70–2. doi: 10.1111/jns.12253 29455455

[60] Shimizu F Koga M Mizukami Y Watanabe K Sato R Takeshita Y . Small nuclear ribonucleoprotein autoantibody associated with blood-nerve barrier breakdown in Guillain-Barré syndrome. Neurol Neuroimmunol Neuroinflamm. (2025) 12:e200405. doi: 10.1212/NXI.0000000000200405 40388674 PMC12092546

[61] Brettschneider J Claus A Kassubek J Tumani H . Isolated blood-cerebrospinal fluid barrier dysfunction: prevalence and associated diseases. J Neurol. (2005) 252:1067–73. doi: 10.1007/s00415-005-0817-9 15789126

[62] Reiber H . Blood-cerebrospinal fluid (CSF) barrier dysfunction means reduced CSF flow not barrier leakage - conclusions from CSF protein data. Arq Neuropsiquiatr. (2021) 79:56–67. doi: 10.1590/0004-282X-anp-2020-0094 33656113

[63] Gonzalez-Quevedo A Carriera RF O’Farrill ZL Luis IS Becquer RM Luis Gonzalez RS . An appraisal of blood-cerebrospinal fluid barrier dysfunction during the course of Guillain Barré syndrome. Neurol India. (2009) 57:288–94. doi: 10.4103/0028-3886.53282 19587469

[64] Ren J Wang J Liu R Guo J Yao Y Hao H . CSF oligoclonal bands in neurological diseases besides CNS inflammatory demyelinating disorders. BMJ Neurol Open. (2025) 7:e000912. doi: 10.1136/bmjno-2024-000912 40051552 PMC11883879

[65] Matà S Galli E Amantini A Pinto F Sorbi S Lolli F . Anti-ganglioside antibodies and elevated CSF IgG levels in Guillain-Barré syndrome. Eur J Neurol. (2006) 13:153–60. doi: 10.1111/j.1468-1331.2006.01161.x 16490046

[66] Ferraro D Galli V Simone AM Bedin R Vitetta F Merelli E . Cerebrospinal fluid anti-Epstein-Barr virus specific oligoclonal IgM and IgG bands in patients with clinically isolated and Guillain-Barré syndrome. J Neurovirol. (2017) 23:329–34. doi: 10.1007/s13365-016-0493-9 27878471

[67] Ruiz M Puthenparampil M Campagnolo M Castellani F Salvalaggio A Ruggero S . Oligoclonal IgG bands in chronic inflammatory polyradiculoneuropathies. J Neurol Neurosurg Psychiatry. (2021) 92:969–74. doi: 10.1136/jnnp-2020-325868 33850000

[68] Sumner RC Parton A Nowicky AV Kishore U Gidron Y . Hemispheric lateralisation and immune function: a systematic review of human research. J Neuroimmunol. (2011) 240-241:1–12. doi: 10.1016/j.jneuroim.2011.08.017 21924504

[69] Duyn JH Schenck J . Contributions to magnetic susceptibility of brain tissue. NMR BioMed. (2017) 30(4):e3546. doi: 10.1002/nbm.3546 27240118 PMC5131875

