# Supplementary material for: Multimodal MRI links gray matter volume loss and temporal lobe susceptibility to CSF immunological profiles in Guillain–Barré syndrome
Source: Front Immunol. 2026 Sep 3;17:1942893. doi: 10.3389/fimmu.2026.1942893 (PMC13582632; doi:10.3389/fimmu.2026.1942893)
Supplement: Supplementary file 1 [file Table1.docx]

**Supplementary Tables. Sensitivity analyses**

**Supplementary Table S1. Brain regions showing altered gray matter volume and QSM values after additional adjustment for diabetes and hypertension**

| Modality | Direction | Cluster | Brain region (AAL3) | Hemisphere | Cluster size, voxels | Peak MNI coordinates (x, y, z) | Peak statistic |
| --- | --- | --- | --- | --- | --- | --- | --- |
| VBM | GBS < HC | 1 | Hippocampus_L / Amygdala_L / ParaHippocampal_L / Temporal_Pole_Sup_L / Olfactory_L / Insula_L / Frontal_Inf_Orb_L / Fusiform_L | Left | 1543 | -21, 3, -19.5 | 5.7338 |
| VBM | GBS < HC | 2 | Hippocampus_R / ParaHippocampal_R / Amygdala_R | Right | 825 | 21, -9, -13.5 | 4.3383 |
| QSM | GBS > HC | 1 | Temporal_Pole_Sup_L / Temporal_Mid_L | Left | 328 | -45, 7, -18 | 5.0356 |
| QSM | GBS > HC | 2 | Fusiform_R / Temporal_Pole_Mid_R / ParaHippocampal_R / Temporal_Inf_R | Right | 296 | 35, 0, -33 | 5.8210 |
| QSM | GBS > HC | 3 | Temporal_Inf_R / Temporal_Mid_R | Right | 347 | 44, -3, -29 | 6.0485 |

**Note:** Only brain regions labeled according to the AAL3 atlas are listed. Sensitivity analysis 2 was performed with additional adjustment for diabetes and hypertension. For VBM analyses, age, sex, total intracranial volume, diabetes, and hypertension were included as covariates. For QSM analyses, age, sex, diabetes, and hypertension were included as covariates. GBS, Guillain-Barre syndrome; HC, healthy controls; VBM, voxel-based morphometry; QSM, quantitative susceptibility mapping; AAL3, Automated Anatomical Labeling atlas 3; MNI, Montreal Neurological Institute.

**Supplementary Table S2. Brain regions showing altered gray matter volume and QSM values after excluding patients with diabetes**

| Modality | Direction | Cluster | Brain region (AAL3) | Hemisphere | Cluster size, voxels | Peak MNI coordinates (x, y, z) | Peak statistic |
| --- | --- | --- | --- | --- | --- | --- | --- |
| VBM | GBS < HC | 1 | Amygdala_L / Hippocampus_L / Temporal_Pole_Sup_L / ParaHippocampal_L / Olfactory_L / Frontal_Inf_Orb_L / Insula_L | Left | 803 | -21, 3, -21 | 5.7937 |
| QSM | GBS < HC | 1 | Putamen_L / Insula_L | Left | 352 | -30, 3, 9 | 4.6594 |
| QSM | GBS > HC | 1 | Fusiform_R / Temporal_Inf_R / Temporal_Mid_R / ParaHippocampal_R / Temporal_Pole_Mid_R | Right | 503 | 43, -3, -30 | 5.2839 |

**Note:** Only brain regions labeled according to the AAL3 atlas are listed. Sensitivity analysis 1 was performed after excluding patients with diabetes, resulting in 19 patients with GBS and 26 healthy controls. GBS, Guillain-Barre syndrome; HC, healthy controls; VBM, voxel-based morphometry; QSM, quantitative susceptibility mapping; AAL3, Automated Anatomical Labeling atlas 3; MNI, Montreal Neurological Institute.

**Supplementary Table S3. Sensitivity analysis of correlations between VBM-ROI gray matter volume and CSF markers**

| ROIs | CSF markers | Spearman ρ | Raw P | FDR-adjusted q |
| --- | --- | --- | --- | --- |
| Cluster 1 | CSF IgA | -0.676 | 0.004* | 0.016* |
| Cluster 1 | CSF IgG | -0.688 | 0.003* | 0.016* |
| Cluster 1 | CSF IgM | -0.501 | 0.048* | 0.064 |
| Cluster 1 | CSF-WBC | -0.664 | 0.005* | 0.016* |
| Cluster 1 | CSF-to-blood glucose ratio | 0.341 | 0.196 | 0.214 |
| Cluster 1 | CSF-to-serum total protein ratio | -0.576 | 0.019* | 0.029* |
| Cluster 2 | CSF IgA | -0.612 | 0.012* | 0.024* |
| Cluster 2 | CSF IgG | -0.662 | 0.005* | 0.016* |
| Cluster 2 | CSF IgM | -0.469 | 0.067 | 0.080 |
| Cluster 2 | CSF-WBC | -0.618 | 0.011* | 0.024* |
| Cluster 2 | CSF-to-blood glucose ratio | 0.188 | 0.485 | 0.485 |
| Cluster 2 | CSF-to-serum total protein ratio | -0.591 | 0.016* | 0.027* |

**Note:** Sensitivity correlation analyses were performed using Spearman rank correlation in the remaining 16 patients with GBS after excluding four patients with CSF WBC counts >50 × 10⁶/L. Cluster 1 corresponds to the left hippocampal/amygdala/parahippocampal cluster, and Cluster 2 corresponds to the right hippocampal/parahippocampal/amygdala cluster. Raw P values <0.05 and FDR-adjusted q values <0.05 are marked with an asterisk (*). Multiple comparisons were corrected using the Benjamini–Hochberg false discovery rate (FDR) procedure across the 12 prespecified comparisons, with q <0.05 considered statistically significant.

Supplementary Figure Legends

Supplementary Figure S1. Associations between mean GMV in the left hippocampal cluster (Cluster 1) and CSF markers after excluding four patients with CSF WBC counts >50 × 10⁶/L (n = 16). Panels show (A) CSF IgA, (B) CSF IgG, (C) CSF WBC count, and (D) the CSF-to-serum total protein ratio. Spearman’s ρ and raw P values are shown; FDR-adjusted q values are provided in Supplementary Table S3.

Supplementary Figure S2. Associations between mean GMV in the right hippocampal cluster (Cluster 2) and CSF markers after excluding four patients with CSF WBC counts >50 × 10⁶/L (n = 16). Panels show (A) CSF IgA, (B) CSF IgG, (C) CSF WBC count, and (D) the CSF-to-serum total protein ratio. Spearman’s ρ and raw P values are shown; FDR-adjusted q values are provided in Supplementary Table S3.
